# Efficient and Accurate Construction of Genetic Linkage Maps from the Minimum Spanning Tree of a Graph

**DOI:** 10.1371/journal.pgen.1000212

**Published:** 2008-10-10

**Authors:** Yonghui Wu, Prasanna R. Bhat, Timothy J. Close, Stefano Lonardi

**Affiliations:** 1 Department of Computer Science and Engineering, University of California Riverside, Riverside, California, United States of America; 2 Department of Botany and Plant Sciences, University of California Riverside, Riverside, California, United States of America; Princeton University, United States of America

## Abstract

Genetic linkage maps are cornerstones of a wide spectrum of biotechnology applications, including map-assisted breeding, association genetics, and map-assisted gene cloning. During the past several years, the adoption of high-throughput genotyping technologies has been paralleled by a substantial increase in the density and diversity of genetic markers. New genetic mapping algorithms are needed in order to efficiently process these large datasets and accurately construct high-density genetic maps. In this paper, we introduce a novel algorithm to order markers on a genetic linkage map. Our method is based on a simple yet fundamental mathematical property that we prove under rather general assumptions. The validity of this property allows one to determine efficiently the correct order of markers by computing the minimum spanning tree of an associated graph. Our empirical studies obtained on genotyping data for three mapping populations of barley (*Hordeum vulgare*), as well as extensive simulations on synthetic data, show that our algorithm consistently outperforms the best available methods in the literature, particularly when the input data are noisy or incomplete. The software implementing our algorithm is available in the public domain as a web tool under the name MSTmap.

## Author Summary

Genetic linkage maps are cornerstones of a wide spectrum of biotechnology applications. In recent years, new high-throughput genotyping technologies have substantially increased the density and diversity of genetic markers, creating new algorithmic challenges for computational biologists. In this paper, we present a novel algorithmic method to construct genetic maps based on a new theoretical insight. Our approach outperforms the best methods available in the scientific literature, particularly when the input data are noisy or incomplete.

## Introduction

Genetic linkage mapping dates back to the early 20th century when scientists began to understand the recombinational nature and cellular behavior of chromosomes. In 1913 Sturtevant studied the first genetic linkage map of chromosome X of *Drosophila melanogaster* [1]. Genetic linkage maps began with just a few to several tens of phenotypic markers obtained one by one by observing morphological and biochemical variations of an organism, mainly following mutation. The introduction of DNA-based markers such as restriction fragment length polymorphism (RFLP), randomly amplified polymorphic DNA (RAPD), simple sequence repeats (SSR) and amplified fragment length polymorpshim (AFLP) caused genetic maps to become much more densely populated, generally into the range of several hundred to more than a thousand markers per genome. More recently, the number of markers has surged well above 1,000 in a number of organisms with the adoption of DArT, SFP and especially SNP markers, the latter providing avenues to 100,000 s to millions of markers per genome. In plants, one of the most densely populated maps is that of *Brassica napus* [2], which was developed from an initial set of 13,551 markers. High density genetic maps facilitate many biological studies including map-based cloning, association genetics and marker assisted breeding. Because they do not require whole genome sequencing and require relatively small expenditures for data acquisition, high density genetic linkage maps are currently of great interest.

A genetic map usually is built using input data composed of the states of *loci* in a set of meiotically derived individuals obtained from controlled crosses. When an order of the markers is computed from the data, the recombinational distance is also estimated. To characterize the quality of an order, various objective functions have been proposed, e.g., *minimum Sum of Square Errors* (SSE) [3], *minimum number of recombination events* (COUNT) [4], *Maximum Likelihood* (ML) [5], *Modified Maximum Likelihood* (MML) [6] which tries to incorporate the presence of possible genotype errors into the ML model, *maximum Sum of adjacent LOD scores* (SALOD) [7], *minimum Sum of Adjacent Recombination Fractions* (SARF) [8], *minimum Product of Adjacent Recombination Fractions* (PARF) [9]. Searching for an optimal order with respect to any of these objective functions is computationally difficult. Enumerating all the possible orders quickly becomes infeasible because the total number of distinct orders is proportional to *n*!, which can be very large even for a small number *n* of markers.

The connection between the traveling salesman problem and a variety of genomic mapping problem is well known, e.g., for the physical mapping problem [10],[11], the genetic mapping problem [12],[13] and the radiation hybrid ordering problem [14]. Various searching heuristics that were originally developed for the traveling salesman problem, such as *simulated annealing* [15], *genetic algorithms* [16], *tabu search* [17],[18], *ant colony optimization*, and iterative heuristics such as *K-opt* and *Lin-Kernighan heuristic* [19] have been applied to the genetic mapping problem in various computational packages. For example, JoinMap [5] and Tmap [6] implement simulated annealing, Carthagene [12],[20] uses a combination of simulated annealing, tabu search and genetic algorithms, AntMap [21] exploits the ant colony optimization heuristic, [22] is based on genetic algorithms, and [23] takes advantage of evolutionary algorithms. Finally, Record [4] implements a combination of greedy and Lin-Kernighan heuristics.

Most of the algorithms proposed in the literature for genetic linkage mapping find reasonably good solutions. Nonetheless, they fail to identify and exploit the combinatorial structures hidden in the data. Some of them simply start to explore the space of the solutions from a purely random order (see, e.g., [12],[23],[5],[21]), while others start from a simple greedy solution (see, e.g., [4],[3]). Here we show both theoretically and empirically that when the data quality is high, the optimal order can be identified very quickly by computing a minimal spanning tree of the graph associated with the genotyping data. We also show that when the genotyping data is noisy or incomplete, our algorithm consistently constructs better genetic maps than the best available tools in the literature. The software implementing our algorithm is currently available as a web tool under the name MSTmap.

## Materials and Methods

We are concerned with genetic markers in the form of *single nucleotide polymorphism* (SNP), more specifically biallelic SNPs. By convention, the two alternative allelic states are denoted as A and B respectively. The organisms considered here are diploids with two copies of each chromosome, one from the mother and the other from the father. A SNP locus may exist in the *homozygous* state if the two allele copies are identical, and in the *heterozygous* state otherwise.

Various population types have been studied in association with genetic mapping, which includes *Back Cross* (BC1), *Doubled Haploid* (DH), *Haploid* (Hap), *Recombinant Inbred Line* (RIL), *advanced RIL*, etc. Our algorithm can handle all of the aforementioned population types. For the sake of clarity, in what follows we will concentrate on the DH population (see the section on barley genotyping data for details on DH populations). The application of our method to Hap, advanced RIL and BC1 populations is straightforward. In Supplementary Text S1, we will discuss the extension of our method to the RIL population (see, e.g., [24] for an introduction to RIL populations).

Building a genetic map is a three-step process. First, one has to partition the markers into *linkage groups*, each of which usually corresponds to a chromosome (sometimes multiple linkage groups can reside on the same chromosome if they are far apart). More specifically, a *linkage group* is defined as a group of loci known to be physically connected, that is, they tend to act as a single group (except for recombination of alleles by crossing-over) in meiosis instead of undergoing independent assortment. The problem of assigning markers to linkage groups is essentially a clustering problem. Second, given a set of markers in the same linkage group, one needs to determine their correct order. Third, the genetic distances between adjacent markers have to be estimated. Before we describe the algorithmic details, the next section is devoted to a discussion on the input data and our optimization objectives.

### Genotyping Data and Optimization Objective Functions

The doubled haploid individuals (a set collectively denoted by *N*) are genotyped on the set *M* of markers, i.e., the state of each marker is determined. The genotyping data are collected into a matrix 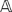 of size *m*×*n*, where *m* = |*M*| and *n* = |*N*|. Each entry in 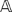 corresponds to a marker and individual pair, which is also called an *observation*. Due to how DH mapping populations are produced (please refer to section on barley genotyping data for details), each observation can exist in two alternative states, namely homozygous A or homozygous E, which are denoted as A and B respectively. The case where there is missing data will be discussed later in this manuscript.

For a pair of markers *l*_1_, *l*_2_ ∈ *M* and an individual *c* ∈ *N*, we say that *c* is a *recombinant* with respect to *l*_1_ and *l*_2_ if *c* has genotype A on *l*_1_ and genotype B on *l*_2_ (or vice versa). If *l*_1_ and *l*_2_ are in the same linkage group, then a recombinant is produced if an odd number of crossovers occurred between the paternal chromosome and the maternal chromosome within the region spanned by *l*_1_ and *l*_2_ during meiosis. We denote with **P** *_i,j_* the probability of a recombinant event with respect to a pair of markers (*l_i_*,*l_j_*). **P** *_i,j_* varies from 0.0 to 0.5 depending on the distance between *l_i_* and *l_j_* At one extreme, if *l_i_* and *l_j_* belong to different LGs, then **P** *_i,j_* = 0.5 because alleles at *l_i_* and *l_j_* are passed down to next generation independently from each other. At the other extreme, when the two markers *l_i_* and *l_j_* are so close to each other that no recombination can occur between them, then **P** *_i,j_* = 0.0. Let (*l_i_*,*l_j_*) and (*l_p_*,*l_q_*) be two pairs of markers on the same linkage group. We say that the pair (*l_i_*,*l_j_*) is *enclosed* in the pair (*l_p_*,*l_q_*) if the region of the chromosome spanned by *l_i_* and *l_j_* is fully contained in the region spanned by *l_p_* and *l_q_*. A fundamental law in genetics is that if (*l_i_*,*l_j_*) is enclosed in (*l_p_*,*l_q_*) then **P** *_i,j_*≤**P** *_p,q_*.

As mentioned in the Introduction, a wide variety of objective functions have been proposed in the literature to capture the quality of the order (SSE, COUNT, ML, MML, SALOD, SARF, PARF, etc.). With the exception of SSE and MML, the rest of the objective functions listed above can be decomposed into a simple sum of terms involving only pairs of markers. Thus, we introduce a weight function *w*: *M*×*M*→ℜ to be defined on pairs of markers. The function *w* is said to be *semi-linear* if *w*(*i*, *j*)≤*w*(*p*, *q*) for all (*l_i_*,*l_j_*) enclosed in (*l_p_*,*l_q_*). For example, if we have three markers in order {*l*_1_,*l*_2_,*l*_3_} and an associated weight function *w* that satisfies semi-linearity, we have *w*(1,3)≥*w*(1,2) and *w*(1,3)≥*w*(2,3) since (*l*_1_,*l*_2_) and (*l*_2_,*l*_3_) are enclosed in (*l*_1_,*l*_3_), but it is not necessary the case that *w*(1,3) = *w*(1,2)+*w*(2,3). The concept of semi-linearity is essential for the development of our marker ordering algorithm as explained below.

For example, the function *w*(*i*, *j*) = **P** *_i,j_* is semi-linear. Another commonly used weight function is *w_lp_*(*i*, *j*) = log(**P** *_i,j_*). Since the logarithm function is monotone, then *w_lp_*(*i*, *j*) is also semi-linear. A more complicated weight function is *w_ml_*(*i*, *j*) = −[**P** *_i,j_*log(**P** *_i,j_*)+(1−**P** *_i,j_*)log(1−**P** *_i,j_*)]. It is relatively easy to verify that *w_ml_*(*i*, *j*) is a monotonically increasing function of **P** *_i,j_* when 0≤**P** *_i,j_*≤0.5, and therefore *w_ml_* is also semi-linear. Observe that all these weight functions are functions in **P** *_i,j_*. Although the precise value of **P** *_i,j_* is unknown, we can compute their estimates from the total number of recombinants in the input genotyping data. For DH populations, the total number of recombinants in *N* with respect to the pair (*l_i_*,*l_j_*) can be easily determined by computing the number *d_i_*_,*j*_ of positions in which row 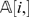 and row 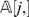 do not match, which corresponds to the *Hamming distance* between 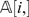 and 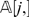. It is easy to prove that *d_i_*_,*j*_/*n* corresponds to the *maximum likelihood estimate* (MLE) for **P** *_i,j_*. When we replace **P** *_i,j_* by its maximum likelihood estimate *d_i_*_,*j*_/*n*, we obtain the following approximate weight functions: *w_p_*′(*i*, *j*) = *d_i_*_,*j*_/*n*, *w_lp_*′ (*i*, *j*) = log(*d_i_*_,*j*_/*n*), and 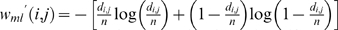.

Our optimization objective is to identify a minimum weight traveling salesman path with respect to either of the aforementioned approximated weight functions, which will be discussed in further details below. We should mention that if *w_p_*′ is used as the weight function, then our optimization objective is equivalent to the SARF or COUNT objective functions (up to a constant). If instead *w_lp_*′ is used, then our optimization objective is equivalent to the logarithm of the PARF objective function (up to a constant). Lastly, if *w_ml_*′ is employed, our objective function is equivalent to the negative of the logarithm of the ML objective function as being employed in [3],[5],[12],[20] (again, up to a constant). Unless otherwise noted, *w_p_*′ is the objective function being employed in the rest of this paper. The experimental results will show that the specific choice of objective function does not have a significant impact on the quality of the final map. In particular, both functions *w_p_*′ and *w_ml_*′ produce very accurate final maps.

### Clustering Markers into Linkage Groups

First observe that when two markers *l_i_* and *l_j_* belong to two different linkage groups, then **P** *_i,j_* = 0.5 and consequently *d_i_*_,*j*_ will be large with high probability. More precisely, let *l_i_* and *l_j_* be two markers that belong to two different LGs, and let *d_i_*_,*j*_ be the Hamming distance between 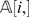 and 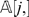. Then, 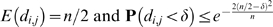 where *δ*<0.5. The proof of this bound can be found in Supplementary Text S1.

In order to cluster the markers into linkage groups, we construct a complete graph *G*(*M*, *E*) over the set of all markers. We set the weight of an edge (*l_i_*, *l_j_*) ∈ *E* to the pairwise distance *d_i_*_,*j*_ between *l_i_* and *l_j_*. As shown in Theorem 1 of Supplementary Text S1, if two markers belong to different LGs, then the distance between them will be large with high probability. Once a small probability *
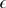
* is chosen by the user (default is *
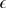
* = 0.00001, in general one should choose *
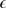
*<0.0001.), we can determine *δ* by solving the equation −2(*n*/2−*δ*)^2^/*n* = log*_e_
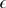
*. We then remove all the edges from *G*(*M*, *E*) whose weight is larger than or equal to *δ*. The resulting graph will break up into connected components, each of which is assigned to a linkage group.

A proper choice of *
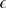
* appears critical in our clustering algorithm. In practice, however, this is not such a crucial issue because the recombinant probability between nearby markers on the same linkage group is usually very small (usually less than 0.05 in dense genetic maps). According to our experience, our algorithm is capable of determining the correct number of LGs for a fairly large range of values of *
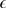
* (see Results and Discussion).

### Ordering Markers in Each Linkage Groups

Let us assume now that all markers in *M* belong to the same linkage group, and that *M* has been preprocessed so that *d_i_*_,*j*_>0 for all *i*, *j* ∈ *M*. The excluded markers for which *d_i_*_,*j*_ = 0 are called *co-segregating* markers, and they identify regions of chromosomes that do not recombine. In practice, we coalesce co-segregating markers into bins, where each bin is uniquely identified by any one of its members. Let *G*(*M*, *E*) be an edge-weighted complete undirected graph on the set of vertices *M*, and let *w* be one of the weight functions defined above. A *traveling salesman path* (TSP) Γ in *G* is a path that visits every marker/vertex once and only once. The weight *w*(Γ) of a TSP Γ is the sum of the weights of the edges on Γ.

The main theoretical insight behind our algorithm is the following. When *w* is semi-linear, the minimum weight TSP of *G* corresponds to the correct order of markers in *M*. Furthermore when the minimum spanning tree (MST) of *G* is unique, the minimum weight TSP of *G* (and thus, the correct order) can be computed by a simple MST algorithm (such as Prim's algorithm). Details of these mathematical facts (with proofs) are given in Supplementary Text S1.

We now turn our attention to the problem of finding a minimum weight TSP in G with respect to one of the approximate weight functions. When the data are clean and *n* is large, the maximum likelihood estimates *d_i_*_,*j*_/*n* will be close to the true probabilities **P** *_i,j_*. Consequently it is reasonable to expect that those approximate weight functions will be also semi-linear, or “almost” semi-linear. Although only in the former case our theory (in particular, Lemma 1 in Supplementary Text S1) guarantees that the minimum weight TSP will correspond to the true order of the markers, in our simulations the order is recovered correctly in most instances. In order to find the minimum weight TSP, we first run Prim's algorithm on *G* to compute the optimum spanning tree, which takes *O*(*n*log*n*). If the MLEs are accurate so that the approximate weight function is semi-linear, our theory (in particular Lemma 2 in Supplementary Text S1) ensures that the MST is a TSP.

**Figure 1:**
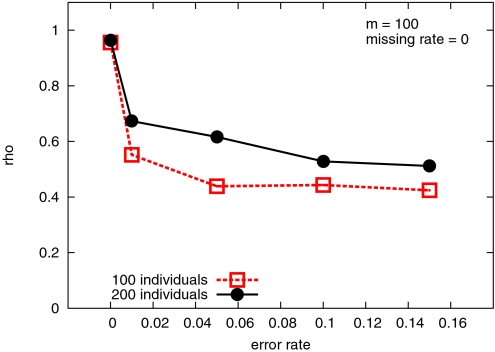
Average *ρ* (rho) for thirty runs on simulated data for several choices of the error rates (and no missing data). The variable *n* represents the number of individuals, and *m* represents the number of markers.

In practice, due to noise in the genotyping data or due to an insufficient number of individuals, the spanning tree may not be a path – but hopefully “very close” to a path. This is exactly what we observed when running MST algorithm on both real data and noise-free simulated data – the MST produced is always “almost” a path. In Results and Discussion we compute the fraction *ρ* of the total number of markers in the linkage group that belong to the longest path of the MST. The closer is *ρ* to 1.0, the closer is the MST to a path. Table 1 on the barley datasets and Figure 1 on simulated data show that *ρ* is always very close to 1.0 when the data is noise-free.

**Table 1:** Summary of the clustering results for the barley data sets.

Data set	# markers (# bins)	# LGs	Sizes of the LGs	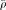
OWB	1562(509)	7	168(65), 235(73), 255(91), 211(60), 278(89), 202(64), 213(67)	0.9978
SM	1270(396)	8	148(49), 217(57), 242(63), 130(49), 225(80), 122(40), 183(57), 3(1)	0.9971
MB	1652(443)	8	215(60), 279(72), 246(77), 141(39), 299(74), 219(54), 248(65), 5(2)	1.0000

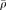
 is the average ρ of the seven largest LGs in each population. The numbers inside the parentheses are the number of bins.

When a tree is not a path, we proceed as follows. First, we find the longest path in the MST, hereafter referred to as the *backbone*. The nodes that do not belong to the path will be first disconnected from it. Then, the disconnected nodes will be re-inserted into the backbone one by one. Each disconnected node is re-inserted at the position which incurs the smallest additional weight to the backbone. The path obtained at the end of this process is our initial solution, which might not be locally optimal.

**Figure 2:**
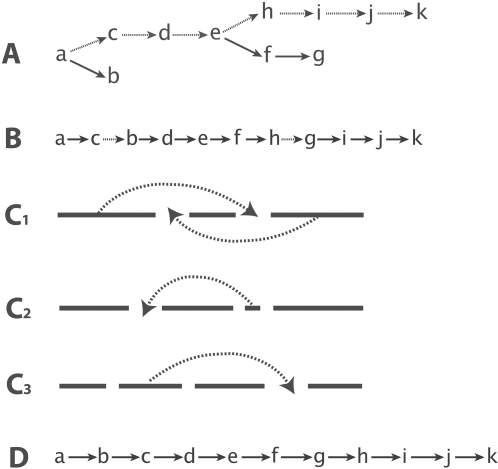
An illustration of the MST-based algorithm. (A) The MST obtained for a synthetic example; the MST is not a TSP yet; the backbone of the MST is shown with dotted edges. (B) An initial TSP obtained from the backbone (see text for details). The dotted edges represent marker pairs in the wrong order. Several local improvement operations are applied to further improve the TSP, namely 2-OPT (C_1_), node-relocation (C_2_) and block-optimize (C_3_). The final TSP is shown in (D).

Once the initial solution is computed, we apply three heuristics that iteratively perform local perturbations in an attempt to improve the current TSP. First, we apply the commonly-used K-opt (*K* = 2 in this case) heuristic. We cut the current path into three pieces, and try all the possible rearrangements of the three pieces. If any of the resulting paths has less total weight, it will be saved. This heuristic is illustrated in Figure 2-C_1_. This procedure is repeated until no further improvement is possible. In the second heuristic, we try to relocate each node in the path to all the other possible positions. If this relocation reduces the weight, the new path will be saved. The second heuristic is illustrated in Figure 2-C_2_.

In our experiments, we observed that K-opt or node relocation may get stuck in local optima if a block of nodes have to be moved as a whole to a different position in order to further improve the TSP. In order to work around this limitation, we designed a third local optimization heuristic, which is called *block-optimize*. The heuristic works as follows. We first partition the current TSP into blocks consisting of consecutive nodes. Let *l*_1_, *l*_2_,…, *l_m_* be the current TSP. We will place *l_i_* and *l_i_*_+1_ in the same block if (1) *w*(*i*, *i*+1)≤*w*(*i*, *j*) for all *i*+1<*j*≤*m* and (2) *w*(*i*, *i*+1)≤*w*(*k*, *i*+1) for all 1≤*i*. Intuitively, the partitioning of the nodes into blocks reflects the fact that the order between the nodes within a block is stable and should be fixed, while the order among the blocks needs to be further explored. After partitioning the current TSP into blocks, we then carry out the K-opt and node relocation heuristics again by treating a block as a single node. The last heuristic, block-optimize, is illustrated in Figure 2-C_3_.

We apply the 2-opt heuristic, the relocation heuristic and the block-optimize heuristic iteratively until none can further reduce the weight of the path. The resulting TSP represents our final solution. A sketch of our ordering algorithm is presented as Algorithm 1 in Supplementary Text S1.

### Dealing with Missing Data

In our discussion so far, we assumed no missing genotypes. This assumption is not very realistic in practice. As it turns out, it is common to have missing data about the state of a marker. Our simulations shows that missing observations do not have as much negative impact on the accuracy of the final map as do genotype errors. Thus, it appears beneficial to leave uncertain genotypes as missing observations rather than arbitrarily calling them one way or the other.

We deal with missing observations via an *Expectation Maximization* (EM) algorithm. Observe that if we knew the order of the markers (or, bins, if we have co-segregating markers), the process of imputing the missing data would be relatively straightforward. For example, suppose we knew that marker *l*_3_ immediately follows marker *l*_2_, and that *l*_2_ immediately follows marker *l*_1_. Let us denote with **P**ˆ*_i,j_* the estimate of the recombinant probabilities between markers *l_i_* and *l_j_*. Let us assume that for an individual *c* the genotype at locus *l*_2_ is missing, but the genotypes at loci *l*_1_ and *l*_3_ are available. Without loss of generality, let us suppose that they are both A. Then, the posterior probability for the genotype at locus *l*_2_ in individual *c* is
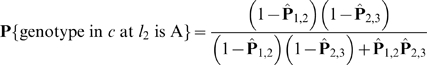
and **P**{genotype in *c* at *l*_2_ is B} = 1−**P**{genotype in *c* at *l*_2_ is A}. This posterior probability is the best estimate for the genotype of the missing observation. Similarly, one can compute the posterior probabilities for different combinations of the genotypes at loci *l*_1_ and *l*_2_.

In order to deal with uncertainties in the data and unify the computation with respect to missing and non-missing observations, we replace each entry in the genotype matrix 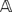 that used to contain symbols A/B with a probability. The probability stored in 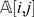 now represents the confidence that we have about marker *l_i_* in individual *c_j_* of being in state A. For the known observations, the probabilities are fixed to be 1 or 0 depending whether the genotype observed is A or B, respectively. The probabilities for the missing observations will be initially set to 0.5.

Our EM algorithm works as follows. We first compute a reasonably good initial order of the markers by ignoring the missing data. To do so, we compute the normalized pairwise distance *d_i_*_,*j*_ as *d_i_*_,*j*_ = *xn*/*n*′, where *n*′ is the number of individuals having non-missing genotypes at both loci *l_i_* and *l_j_*, *x* is the number of individuals having different genotypes at loci *l_i_* and *l_j_* among the *n*′ individuals being considered, and *n* is the total number of individuals. With the normalized pairwise distances, we rely on the function Order (Supplementary Text S1, Algorithm 1) to compute an initial order.

After an initial order has been computed, we iteratively execute an E-step followed by an M-step. In the E-step, given the current order of the markers, we adjust the estimate for a missing observation at marker 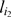 on individual *c_j_* as follows
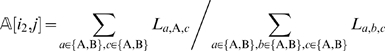
(1)where 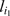 is the marker immediately preceding 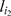 in the most recent ordering, 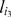 is the marker immediately following 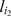, and *L_a_*_,*b*,*c*_ is the likelihood of the event 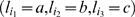 at the three consecutive loci. The right hand side of Equation (1) is simply the posterior probability of the missing observation 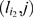 being A. The quantity *L_a_*_,*b*,*c*_ is straightforward to compute. For example, 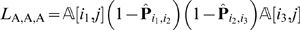, where 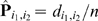, 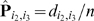 are the MLEs for 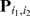 and 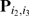 respectively, and 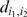 and 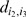 are the pairwise distance computed in the previous M step or the initialization step when the initial order is computed. In the case where the missing observation is at the beginning or at the end of the map, the above estimates must be adjusted slightly.

Following the E-step, we execute an M-step. We need to re-compute the pairwise distances according to the new estimates of the missing data. Given that now 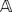 contains probabilities, the expected pairwise distance between two markers *l_i_* and *l_j_* can be computed as follows
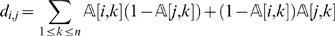
(2)With the updated pairwise distances, we use the function Order again to compute a new order of the markers.

An E-step is followed by another M-step, and this iterative process continues until the marker order converges. In our experimental evaluations, the algorithm converges quickly, usually in less than ten iterations. The pseudo-code for the EM algorithm is presented as Algorithm 2 in Supplementary Text S1.

We should mention that our EM algorithm is significantly different from the EM algorithms employed in MapMaker [25] or Carthagene [12]. The EM algorithms used in MapMaker and Carthagene are not used to determine the order, but rather to estimate the recombination probabilities between adjacent markers in the presence of missing data. In MSTmap, our EM method deals with missing data in a way which is very tightly coupled with the problem of finding the best order of the markers.

### Detecting and Removing Erroneous Data

As commonly observed in the literature (see, e.g., [26],[27]), with conventional mapping software such as JoinMap, Carthagene or Record, the existence of genotyping errors can have a severe impact on the quality of the final maps. With even a relatively small amount of errors, the order of the markers can be compromised. Therefore, it is necessary to detect erroneous genotype data.

In practice, genotype errors do not distribute evenly across markers. Usually a few “bad markers” tend to be responsible for the majority of errors. Removing those bad markers is relatively easy because they will appear isolated from the other markers in terms of Hamming distance *d_i_*_,*j*_. We can simply look for markers which are more than a certain distance (a parameter specified by the user, default is 15 cM) away from all other markers. Bad markers are deleted completely from the dataset.

Residual sources of genotyping errors are more difficult to deal with. Given that in practice missing observations have much less negative impact on the quality of the map than errors, our strategy is to identify suspicious data and treat them as missing observations. When doing so, however, we should be careful not to introduce too many missing observations.

**Figure 3:**
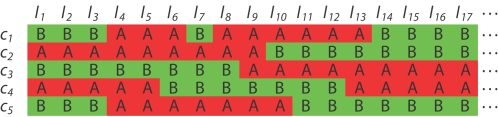
An example of a singleton (double crossover). Each row refers to an individual and each column refers to a marker locus. Given the current order, the entry (*c*_1_, *l*_7_) appears to be a possible error because its state differs from both its immediately preceding and following markers.

In high density genetic mapping, a genotype error usually manifests itself as a *singleton* (or a *double cross-over*) under a reasonably accurate ordering of the markers. A *singleton* is a SNP locus whose state is different from both the SNP marker immediately before and after. An example of a singleton is illustrated in Figure 3. A reasonable strategy to deal with genotyping errors is to iteratively remove singletons by treating them as missing observations and then refine the map by running the ordering algorithm. The problem of this strategy is that at the beginning of this process the number of errors might be high and the marker orders are not very accurate. As a consequence, the identified singletons might be false positives.

We deal with this problem by taking into consideration the neighborhood of a marker instead of just looking at the immediately preceding and following ones. Along the lines of the approach proposed in SMOOTH [26], we define
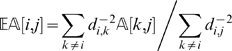
(3)where *i* is a marker and *j* is an individual. The quantity 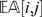 estimates the state of a locus given its distance to other markers (and their states). The estimate is a weighted average of the information from all other markers, and the weight is proportional to the inverse of the square of the distance. One can approximate 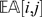 by considering only a small set (say 8) of the closest markers to compute the estimate. When 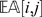 is far from 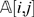, then the observation (*i*, *j*) is regarded as suspicious and is treated as a missing observation. In our implementation, we consider an observation suspicious when 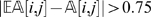.

In our iterative process (1) we detect possible errors using 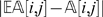, (2) we refine the map by calling Algorithm 3 (Supplementary Text S1), (3) we estimate the missing data and (4) we re-compute the distances *d_i_*_,*j*_ according to Equation (2). The number of iterations should depend on the quality of the data. If the original data are noisy, more iterations are needed. We propose an adaptive approach to dynamically determine when to stop the iterative process. Let *X* be the total number of suspicious observations that have been detected so far plus the total number of cross-overs still present in the latest order. Observe that an error usually result in two cross-overs (refer to Figure 3 for an example). By treating an error as a missing observation, the total number of suspicious observations will increase by one, but the total number of crossovers will decrease by two. Overall, the quantity *X* will decrease by one in the next iteration. On the other hand, if an observation is indeed correct but is mistakenly treated as a missing, *X* will increase by one in the next iteration. Based on this analysis, we stop the iterative process as soon as the quantity *X* begins to increase.

In passing, we should mention that Equation (3) can also be used to estimate missing data. According to our experimental studies, it gives comparable performance to the EM algorithm we proposed in the previous section. Our complete algorithm, which incorporates clustering markers into linkage groups, missing data estimation, and error detection, is presented in Supplementary Text S1 as Algorithm 3. We named our algorithm MSTmap since the initial orders of the markers are inferred from the MST of a graph.

### Computing the Genetic Distances

Mapping functions are used to convert the recombination probabilities *r* to a genetic distance *D* that reflects the actual frequency of crossovers. They correct for undetected double crossovers and cross over interference. The Haldane mapping function [28] assumes that crossovers occur independently and thus do not adjust for interference, while the Kosambi mapping function [29] adjusts for crossover interference assuming that one crossover inhibits another nearby. The Haldane distance function is defined as 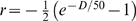, whereas the Kosambi distance function is 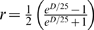. Both functions are defined for 0<*r*<0.5. When the crossover interference is not known, Kosambi should be used by default. If the frequency of crossover is low or in the case of high density maps when the distance between adjacent markers is low, either of them can be safely used [30].

## Results/Discussion

We implemented our algorithm in C++ and carried out extensive evaluations on both real data and simulated data. The software is available in the public domain at the address http://www.cs.ucr.edu/~yonghui/mstmap.html.

The four tools benchmarked here were run on relatively fast computers by contemporary standards. MSTmap was run on a Linux machine with 32 1.6 GHz Intel Xeon processors and 64 GB memory, Carthagene was executed on a Linux machine with a dual-core 2 GHz Intel Xeon processor and 3 GB memory whereas Record and JoinMap were both run on a Windows XP machine with a dual-core 3 GHz Intel Pentium processor and 3 GB of main memory. We had to use different platforms because some of these tools are platform-specific (i.e., Record and JoinMap only run on Windows, Carthagene only runs on Linux). Note that MSTMap was run on the platform with the slowest CPU. The fact that MSTMap was run on a machine with multiple CPUs and large quantity of main memory did not create an unfair advantage. MSTMap is single threaded and thus it exploits only one CPU. The space complexity of MSTMap is *O*(*n* ^2^), where *n* is the number of markers per linkage group. Under our simulation studies, *n* is less than 500, which translates in about of 1 GB memory which is a relatively small amount.

### Barley Genotyping Data

The real genotyping data come from an ongoing genetic mapping project for barley (*Hordeum vulgare*) (see http://barleycap.org/ and http://www.agoueb.org/ for more details on this project). In total we made use of three mapping populations, all of which are DH populations. Doubled haploid (DH) technology refers to the use of microspore or anther culture (ovary culture in some species) to obtain haploid embryos and subsequently double the ploidy level. Briefly, a DH population is prepared as follows. Let *M* be the set of markers of interest. Pick two highly inbred (fully homozygous) parents *p*_1_ and *p*_2_. We assume that the parents *p*_1_ and *p*_2_ are homozygous for every marker in *M* (those markers that are heterozygous in either *p*_1_ or *p*_2_ are simply excluded from consideration), and the same marker always has different allelic states in the two parents (those markers having the same allelic state in both parents are also excluded from *M*). By convention, we use symbol A to denote the allelic states appearing in *p*_1_ and B to denote the allelic states appearing in *p*_2_. Parent *p*_1_ is crossed with parent *p*_2_ to produce the first generation, called *F1*. The individuals in the F1 generation are heterozygous for every marker in *M*, with one chromosome being all A and the other chromosome being all B. Gametes produced by meiosis from the F1 generation are fixed in a homozygous state by doubling the haploid chromosomes to produce a doubled haploid individual. The ploidy level of haploid embryo could be doubled by chemical (example colchicine) treatment to obtain doubled haploid plants with 100% homozygosity. This technology is available in some crops to speed up the breeding procedure (see, e.g., [31]).

The first mapping population is the result of crossing Oregon Wolfe Barley Dominant with Oregon Wolfe Barley Recessive (see http://barleyworld.org/oregonwolfe.php). The Oregon Wolfe Barley (OWB) data set consists of 1,562 markers genotyped on 93 individuals. The second mapping population is the result of a cross between Steptoe with Morex (see http://wheat.pw.usda.gov/ggpages/SxM/), which consists of 1,270 markers genotyped on 92 individuals. It will be referred to as the SM dataset from here on. The third mapping population is the result of a cross between Morex with Barke recently developed by Nils Stein and colleagues at the Leibniz Institute of Plant Genetics and Crop Plant Research (IPK), which contains 1,652 markers on 93 individuals. This latter dataset will be referred to as MB in our discussion. The genotypes of SNPs for the above data sets were determined via an Illumina GoldenGate Assay. Very few of the genotypes are missing. The three mapping populations together contain only 51 missing genotype calls out of the total of 417,745. The three barley data sets are expected to contain seven LGs, one for each of the seven barley chromosomes.

### Synthetic Genotyping Data

The simulated data set is generated according to the following procedure (which is identical to the one used in [4]). First four parameters are chosen, namely the number *m* of markers to place on the genetic map, the number *n* of individuals to genotype, the error rate *η* and the missing rate γ. Following the choice of *m*, a “skeleton” map is produced, according to which the genotypes for the individuals will be generated. The markers on the skeleton map are spaced at a distance of 0.5 centimorgan plus a random distance according to a Poisson distribution. On average, the adjacent markers are 1 centimorgan apart from each other. The genotypes for the individuals are then generated as follows. The genotype at the first marker is generated at random with probability 0.5 of being A and probability 0.5 of being B. The genotype at the next marker depends upon the genotype at the previous marker and the distance between them. If the distance between the current marker and the previous marker is *x* centimorgan, then with probability *x*/100, the genotype at the current locus is the opposite of that at the previous locus, and with probability 1−*x*/100 the two genotypes are the same. Finally, according to the specified error rate and missing rate, the current genotype is flipped to purposely introduce an error or is simply deleted to introduce a missing observation. Following this procedure, various datasets for a wide range of choices of the parameters were generated.

### Evaluation of the Clustering Algorithm

First, we evaluated the effectiveness of our clustering algorithm on the three datasets for barley. Since the genome of barley consists of seven chromosome pairs, we expected the clustering algorithm to produce seven linkage groups. Using the default value for *ε*, our algorithm produced seven linkage groups for the OWB data set, eight linkage groups for the MB data set and eight linkage groups for the SM data set. The same results can be obtained in a rather wide range of values of *
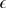
*. For example, for any choice of *
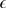
* ∈ [0.000001,0.0001] the OWB data set is always clustered into seven LGs. The smallest linkage group in the SM data set contains three markers in a single bin. The smallest linkage group in the MB data set contains five markers in two bins. By comparing the three maps with each other, we determined that these small isolated linkage groups in the SM and MB populations are at the telomere far away from the rest of the markers on the same chromosome. The result of the clustering algorithm is summarized in Table 1. We also compared our clusters with those produced by JoinMap. The clusters were identical.

### Evaluation of the Quality of the Minimum Spanning Trees

In this second evaluation step, we verified that on real and simulated data, the MSTs produced by MSTmap are indeed very close to TSPs. This experimental evaluation corroborates the fact that the MSTs produced are very good initial solutions. Here, we computed the fraction *ρ* of the total number of bins/vertices in the linkage group that belong to the longest path (backbone) of the MST. The closer is *ρ* to 1, the closer is the MST to a path.

Table 1 shows that on the barley data sets, the average value for *ρ* for the seven linkage groups (not including the smallest LG in the SM data set) is always very close to 1. Indeed, 19 of the 21 MSTs are paths. The remaining 2 MSTs are all very close to paths, with just one node hanging off the backbone. When the MSTs generated by our algorithm are indeed paths, the resulting maps are guaranteed to be optimal, thus increasing the confidence in the correctness of the orders obtained.

On the simulated dataset with no genotyping errors, *ρ* is again close to one (see Figure 1) for both *n* = 100 and *n* = 200 individuals. When the error rate is 1%, the ratio drops sharply to about 0.6. This is due to the fact that the average distance between nearby markers is only one centimorgan. One percent error introduces an additional distance of two centimorgans which is likely to move a marker around in its neighborhood. The value for *ρ* for error rates up to 15% are computed and are presented in Figure 1. At 15% error rate, the backbone contains only about 40% of the markers. However, this relatively short backbone is still very useful in obtaining a good map since it can be thought as a sample of the markers in their true order. Also, observe that increasing the number of individuals will slightly increase the length of the backbone. However, the ratio remains the same irrespective of the number of markers we include on a map (data not shown).

### Evaluation of the Error Detection Algorithm

Third, we evaluated the accuracy and the effectiveness of the error detection algorithm. Synthetic datasets with a known map and various choices of error rate and missing rate were generated. We ran our tool on each dataset, and by comparing the map produced by MSTMap with the true map we collected a set of relevant statistics.

Given a map produced by MSTmap we define a pair of markers to be *erroneous* if their relative order is reversed when compared to the true map. The number *E* of erroneous marker pairs ranges from 0 to *m*(*m*−1)/2, where *m* is the number of markers. We have *E* = 0 when the two maps are identical, and *E* = *m*(*m*−1)/2 when one map is the reverse of the other. Since the orientation of the map is not important in this context, whenever *E*>*m*(*m*−1)/4, one of the maps is flipped and *E* is recomputed. Notice that *E* is more sensitive to global reshuffling than to local reshuffling. For example, assume that the true order is the identity permutation. The value of *E* for the following order 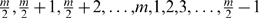 is *m*(*m*−1)/4, whereas *E* for the order 2,1,4,3,6,5,…,*m*,*m*−1 is *m*(*m*−1)/2. For reasonably large *m*, *m*(*m*−1)/2 is much smaller than *m*(*m*−1)/4. The fact that *E* is more sensitive to global reshuffling is a desirable property since biologists are often more interested in the correctness of the global order of the markers than the local order.

The number of erroneous marker pairs conveys the overall quality of the map produced by MSTmap, however *E* depends on the number *m* of markers. The larger is *m*, the larger *E* will be. Sometimes it is useful to normalize *E* by taking the transformation 1−(4*E*/(*m*(*m*−1))). The resulting statistic is essentially the Kendall's *τ* statistic. The *τ* statistic ranges from 0 to 1. The closer is the statistic to 1, the more accurate the map is. We will present the *τ* statistic along with the *E* statistic when it is necessary.

The next three statistics we collected are the percentage of true positives, the percentage of false positives, and the percentage of false negatives, which are denoted as %*t*_*pos*, %*tf*_*pos* and %*f*_*neg* respectively. For each dataset, the list of suspicious observations identified by MSTmap is compared with the list of true erroneous observations that were purposely added when the data was first generated. The value of %*t*_*pos* is the number of suspicious observations that are truly erroneous divided by the total number *nm* of observations. The value %*f* _*pos* is the number suspicious observations that are in fact correct divided by the total number of observations. Likewise, %*f*_*neg* is the number of erroneous observations that are not identified by MSTmap. The three performance metrics are intended to capture the overall accuracy of the error detection scheme. Finally, we collected the running time on each data set.

Table 2 summarizes the statistics for *n* = 100, *m* = 100, when the error rate and the missing rate range from 0% to 15%. An inspection of the table reveals that irrespective of the choice of *η* and γ our error detection scheme is able to detect most of the erroneous observations without introducing too many false positives. When the input data are noisy, the quality of the final maps with error detection is significantly better than those without. However, if the input data are clean (corresponding to rows in the table where *η* = 0), the quality of the maps with error detection deteriorates slightly. Results for other choices of *m* and *n* are presented in Table 4 and Table 5. Similar conclusions can be drawn.

**Table 2:** Summary of the accuracy and effectiveness of our error detection scheme for *m* = 100, *n* = 100 and various choices of *η* and γ.

*n*, *m* = 100					*E*		
γ	*η*	%*f*_*pos*	%*t*_*pos*	%*f*_*neg*	*w_p_*′	*w*′*_ml_*	*w_p_*′ no err.
0.00	0.00	0.00186	0.00000	0.00000	1.50	1.43	1.80
0.00	0.01	0.00441	0.00956	0.00049	15.10	15.80	38.93
0.00	0.05	0.00442	0.04643	0.00357	41.37	42.93	165.50
0.00	0.10	0.00682	0.08754	0.01229	96.53	96.07	468.63
0.00	0.15	0.01086	0.12188	0.02720	221.03	238.77	1187.60
0.01	0.00	0.00177	0.00000	0.00000	1.83	3.27	1.27
0.05	0.00	0.00150	0.00000	0.00000	6.47	6.17	5.23
0.10	0.00	0.00135	0.00000	0.00000	18.07	18.60	9.23
0.15	0.00	0.00124	0.00000	0.00000	16.13	16.40	10.00
0.01	0.01	0.00357	0.00966	0.00050	11.47	11.83	44.20
0.05	0.05	0.00421	0.04305	0.00433	52.90	54.13	144.67
0.10	0.10	0.00631	0.07641	0.01300	140.67	150.40	532.47
0.15	0.15	0.00994	0.09494	0.03277	379.17	353.53	1040.70

Each row in the table is an average of 30 independent runs. The columns *w_p_*′ and *w*′*_ml_* correspond to the number of erroneous marker pairs (*E*) made by MSTmap under the objective function *w_p_*′ and *w*′*_ml_* respectively with error detection. The column “*w_p_*′ no err.” corresponds to the number of erroneous markers pairs made by MSTmap under the objective function *w_p_*′ without error detection.

In Table 2, we also compare the quality of the final maps under different objective functions. The objective functions 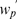 (SARF) and 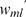 (ML) give very similar results. Similar results are observed for other choices of *n* and *m* (data not shown).

### Evaluation of the Accuracy of the Ordering

In the fourth and final evaluation, we use simulated data to compare our tool against several commonly used tools including JoinMap [5], Carthagene [12] and Record [4]. JoinMap is a commercial software that is widely used in the scientific community. It implements two algorithms for genetic map construction, one is based on regression [3] whereas the other based on maximum likelihood [5]. Our experimental results for JoinMap are obtained with the “maximum likelihood based algorithm” since it is orders of magnitude faster than the “regression based algorithm” (see the manual of JoinMap for more details). Due to the fact that JoinMap is GUI-based (non-scriptable), we were able to collect statistics for only a few datasets. Carthagene and Record on the other hand are both scriptable, which allows us to carry out more extensive comparisons. However, due to the slowness of Carthagene (when *n* = 300, it takes more than several hours to finish), we applied it only to small data sets (*n* = 100). The most complete comparison was carried out between MSTmap and Record.

**Figure 4:**
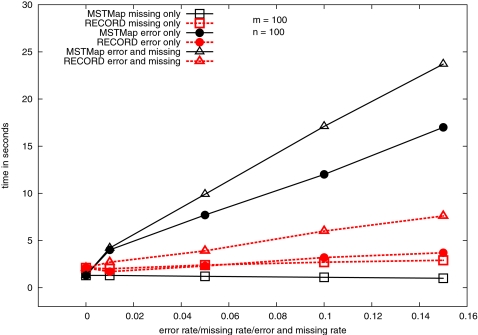
Running time of MSTmap and Record with respect to error rate or missing rate or error and missing rate. Every point in the graph is an average of 30 runs. The lines “missing only” correspond to data sets with no error (*η* = 0, γ is on the *x*-axis). Similarly, lines “error only” correspond to data sets with no missing (γ = 0, *η* is on the *x*-axis), and lines “error and missing” correspond to data sets with equal missing rate and error rate (*η* = γ is on the *x*-axis).

As we have done in the previous subsection, we employ the number of erroneous pairs to compare the quality of the maps obtained by different tools. The results for *n* = 100 and *m* = 100 are summarized in Table 3. A more thorough comparison of MSTmap and Record is presented in Table 4 and Table 5. Several observations are in order. First, MSTmap constructs significantly better maps than the other tools when the input data are noisy. When the data are clean and contain many missing observations (i.e., *η* = 0 and γ is large), Carthagene produces maps which are slightly more accurate than those by MSTmap. However, if we knew the data were clean, by turning off the error-detection in MSTmap we would obtain maps of comparable quality to Carthagene in a much shorter running time. Please refer to the “*w_p_*′ no err” column for the *E* statistics of MSTmap when the error detection feature is turned off. Second, Carthagene appears to be better than Record when the data are clean (*η* = 0). When the data are noisy, Record constructs more accurate maps than Carthagene. Third, MSTmap and Record are both very efficient in terms of running time, and they are much faster than Carthagene. A clearer comparison of the running time between MSTmap and Record is presented in Figure 4. The figure shows that MSTmap is even faster than Record when the data set contains no errors. However as the input data set becomes noisier, the running time for MSTmap also increases. This is because our adaptive error detection scheme needs more iterations to identify erroneous observations, and consequently takes more time. However, this lengthened execution does pay off with a significantly more accurate map. Fourth and last, Table 3, 4 and 5 show that the overall quality of the maps produced by MSTmap is usually very high. In most scenarios, the *τ* statistic is greater than 0.99.

**Table 3:** Comparison between MSTmap, JoinMap, Carthagene and Record for *n* = 100 and *m* = 100.

*n*, *m* = 100		MSTmap		Record		Carthagene		JoinMap	
γ	*η*	*E*	time	*E*	time	*E*	time	*E*	time
0.00	0.00	1.50	1.3	**1.3**	2.1	2.5	255.0	1.7	<60
0.00	0.01	**15.10**	4.0	46.6	1.7	58.2	275.7	**-**	**-**
0.00	0.05	**41.37**	7.7	129.1	2.3	300.3	267.4	**-**	**-**
0.00	0.10	**96.53**	12.0	450.8	3.2	680.0	265.2	**-**	**-**
0.00	0.15	**221.03**	17.0	1064.8	3.7	1378.5	276.8	**-**	**-**
0.01	0.00	**1.83**	1.3	34.7	2.0	2.8	300.1	**-**	**-**
0.05	0.00	6.47	1.2	44.0	2.4	**5.4**	363.1	**-**	**-**
0.10	0.00	18.07	1.1	49.7	2.7	**7.0**	416.6	**-**	**-**
0.15	0.00	16.13	1.0	64.8	2.9	**9.0**	486.2	**-**	**-**
0.01	0.01	**11.47**	4.2	54.8	2.7	49.0	310.0	53.7	<60
0.05	0.05	**52.90**	9.9	164.1	3.9	296.4	368.4	370.2	<60
0.10	0.10	**140.67**	17.1	683.4	6.0	837.0	430.2	**-**	**-**
0.15	0.15	**379.17**	23.7	1387.4	7.6	1273.3	500.1	**-**	**-**

Each number presented in the table is averaged over 30 independent runs, except for those of JoinMap, which are averaged over 10 independent runs. Column *E* reports the average number of erroneous marker pairs. The running time is reported as number of seconds.

**Table 4:** Comparison between MSTmap and Record for *m* = 300,500 and *n* = 100.

		MSTmap			MSTMap no err. detection			Record		
γ	*η*	*E*	*τ*	time	*E*	*τ*	time	*E*	*τ*	time
*M* = 300, *n* = 100										
0.00	0.00	**5.2**	0.9998	12.1	6.3	0.9997	11.4	6.6	0.9997	25.7
0.00	0.01	**42.7**	0.9981	39.0	135.0	0.9940	29.1	135.5	0.9940	17.5
0.00	0.05	**136.9**	0.9939	80.1	407.9	0.9818	55.4	423.0	0.9811	23.3
0.00	0.10	**338.1**	0.9849	147.7	1104.5	0.9507	67.9	2946.3	0.8686	26.3
0.00	0.15	**612.0**	0.9727	221.0	5662.3	0.7475	78.8	8202.9	0.6342	31.9
0.01	0.00	**6.1**	0.9997	13.5	6.7	0.9997	11.5	107.5	0.9952	19.6
0.05	0.00	19.6	0.9991	12.8	**14.4**	0.9994	10.6	133.0	0.9941	25.4
0.10	0.00	34.8	0.9984	13.1	**17.7**	0.9992	10.0	156.8	0.9930	28.8
0.15	0.00	52.1	0.9977	11.5	**30.3**	0.9986	8.4	197.4	0.9912	32.3
0.01	0.01	**32.6**	0.9985	41.1	134.4	0.9940	31.6	153.5	0.9932	26.6
0.05	0.05	**150.6**	0.9933	114.1	399.1	0.9822	73.5	510.7	0.9772	34.7
0.10	0.10	**402.0**	0.9821	228.1	1089.9	0.9514	84.2	3626.1	0.8383	42.4
0.15	0.15	**757.8**	0.9662	356.7	5605.2	0.7500	95.6	10970.7	0.5108	54.5
*m* = 500, *n* = 100										
0.00	0.00	**8.6**	0.9999	33.6	10.6	0.9998	32.4	10.4	0.9998	32.5
0.00	0.01	**68.8**	0.9989	105.4	219.8	0.9965	83.4	233.5	0.9963	57.0
0.00	0.05	**207.4**	0.9967	239.5	663.5	0.9894	171.6	1308.4	0.9790	75.3
0.00	0.10	**532.1**	0.9915	467.6	2226.6	0.9643	198.8	9797.2	0.8429	78.8
0.00	0.15	**1014.9**	0.9837	698.7	9652.8	0.8452	247.7	32850.8	0.4733	90.2
0.01	0.00	**11.5**	0.9998	32.4	11.9	0.9998	32.2	183.3	0.9971	60.2
0.05	0.00	30.0	0.9995	29.2	**20.6**	0.9997	28.7	225.6	0.9964	84.4
0.10	0.00	63.4	0.9990	27.4	**34.6**	0.9994	27.0	249.1	0.9960	95.8
0.15	0.00	86.2	0.9986	24.7	**55.8**	0.9991	24.4	312.0	0.9950	104.1
0.01	0.01	**53.7**	0.9991	106.5	224.8	0.9964	91.9	238.3	0.9962	81.4
0.05	0.05	**238.0**	0.9962	349.3	639.6	0.9897	234.5	4794.9	0.9231	99.0
0.10	0.10	**629.0**	0.9899	739.0	1694.7	0.9728	291.0	23968.4	0.6157	121.6
0.15	0.15	**1256.5**	0.9799	1256.0	8501.9	0.8637	267.0	37382.9	0.4007	162.0

The columns under “MSTMap no err. detection” contains results obtained when running MSTmap with no error detection. We report Kendall's *τ* statistic and the number *E* of erroneous marker pairs. The running time is reported in seconds.

**Table 5:** Comparison between MSTmap and Record for *m* = 100,300,500 and *n* = 200.

		MSTmap			MSTMap no err. detection			Record		
γ	*η*	*E*	*τ*	time	*E*	*τ*	time	*E*	*τ*	time
*M* = 100, *n* = 200										
0.00	0.00	3.2	0.9987	4.0	0.8	0.9997	3.9	**0.5**	0.9998	4.4
0.00	0.01	**6.7**	0.9973	8.3	19.1	0.9923	6.3	18.4	0.9926	3.8
0.00	0.05	**19.1**	0.9923	12.2	65.5	0.9735	7.2	55.6	0.9775	5.0
0.00	0.10	**58.5**	0.9764	17.1	192.3	0.9223	8.4	196.0	0.9208	6.5
0.00	0.15	**112.0**	0.9548	22.6	533.0	0.7847	9.1	513.9	0.7924	7.9
0.01	0.00	2.3	0.9991	4.2	**0.6**	0.9998	3.8	16.9	0.9932	4.0
0.05	0.00	5.3	0.9979	3.8	**1.2**	0.9995	3.6	19.2	0.9922	4.5
0.10	0.00	11.2	0.9955	3.4	**2.0**	0.9992	3.2	22.7	0.9908	5.2
0.15	0.00	11.2	0.9955	3.2	**3.2**	0.9987	3.0	26.1	0.9895	5.6
0.01	0.01	**4.5**	0.9982	8.4	19.9	0.9919	6.5	22.9	0.9908	4.9
0.05	0.05	**25.2**	0.9898	15.6	67.8	0.9726	7.7	91.1	0.9632	10.3
0.10	0.10	**71.2**	0.9712	24.8	171.8	0.9306	8.5	293.2	0.8815	13.5
0.15	0.15	**138.2**	0.9442	40.0	587.0	0.7628	9.3	1020.9	0.5875	13.2
*m* = 300, *n* = 200										
0.00	0.00	8.2	0.9996	38.1	**1.9**	0.9999	34.9	2.4	0.9999	98.1
0.00	0.01	**19.7**	0.9991	75.8	59.1	0.9974	58.1	59.6	0.9973	35.7
0.00	0.05	**64.4**	0.9971	111.8	188.3	0.9916	73.5	186.6	0.9917	49.0
0.00	0.10	**169.3**	0.9925	187.0	525.8	0.9766	93.9	534.0	0.9762	55.2
0.00	0.15	**328.2**	0.9854	254.3	1350.3	0.9398	94.0	2269.8	0.8988	63.5
0.01	0.00	8.5	0.9996	35.4	**1.8**	0.9999	34.9	48.3	0.9978	38.3
0.05	0.00	15.9	0.9993	32.4	**3.8**	0.9998	31.7	54.1	0.9976	43.1
0.10	0.00	28.4	0.9987	29.6	**8.9**	0.9996	29.0	62.5	0.9972	49.6
0.15	0.00	38.2	0.9983	27.5	**12.2**	0.9995	26.8	81.0	0.9964	55.4
0.01	0.01	**16.7**	0.9993	75.7	63.5	0.9972	61.0	66.1	0.9971	45.6
0.05	0.05	**66.9**	0.9970	146.9	194.0	0.9914	84.3	227.3	0.9899	63.9
0.10	0.10	**205.7**	0.9908	281.3	517.4	0.9769	96.8	758.6	0.9662	82.3
0.15	0.15	**418.5**	0.9813	457.8	1179.5	0.9474	113.5	5464.8	0.7563	104.7
*m* = 500, *n* = 200										
0.00	0.00	12.1	0.9998	99.0	**2.8**	1.0000	97.1	4.2	0.9999	123.9
0.00	0.01	**32.3**	0.9995	206.7	100.1	0.9984	162.1	100.0	0.9984	113.8
0.00	0.05	**104.1**	0.9983	295.5	282.5	0.9955	223.2	323.6	0.9948	149.9
0.00	0.10	**291.3**	0.9953	495.0	810.5	0.9870	287.8	1011.1	0.9838	158.7
0.00	0.15	**542.5**	0.9913	772.4	2099.5	0.9663	323.0	11335.7	0.8183	184.6
0.01	0.00	15.1	0.9998	96.9	**3.3**	0.9999	95.5	79.8	0.9987	120.9
0.05	0.00	28.7	0.9995	90.1	**6.0**	0.9999	88.4	88.6	0.9986	133.5
0.10	0.00	47.4	0.9992	83.0	**10.7**	0.9998	81.3	107.1	0.9983	146.5
0.15	0.00	56.5	0.9991	77.0	**17.7**	0.9997	75.5	123.1	0.9980	167.2
0.01	0.01	**30.2**	0.9995	216.0	98.4	0.9984	172.7	183.8	0.9971	143.9
0.05	0.05	**112.0**	0.9982	463.0	331.4	0.9947	260.9	632.2	0.9899	193.2
0.10	0.10	**342.1**	0.9945	852.5	940.9	0.9849	349.4	2110.1	0.9662	226.1
0.15	0.15	**725.9**	0.9884	1506.2	2190.2	0.9649	368.7	15200.3	0.7563	274.5

The columns under “MSTMap no err. detection” contains results obtained when running MSTmap with no error detection. We report Kendall's *τ* statistic and the number *E* of erroneous marker pairs. The running time is reported in seconds.

An extensive comparison of MSTmap and Record for other choices of *m* and *n* is presented in Table 4 and Table 5. Notice that even without error detection, MSTmap is more accurate than Record.

We have also compared MSTmap, Record, JoinMap and Carthagene on real genotyping data for the barley project. We carried out several rounds of cleaning the input data after inspecting the output from MSTmap (in particular, we focused on the list of suspicious markers and genotype calls reported by MSTmap), then the data set was fed into MSTmap, Record, JoinMap and Carthagene. The results show that the genetic linkage maps obtained by MSTmap and JoinMap are identical in terms of marker orders. MSTmap, Carthagene and Record differ only at the places where there are missing observations. At those locations, MSTmap groups markers in the same bin, while Carthagene and Record split them into two or more bins (at a very short distance, usually less than 0.1 cm).

### Conclusion

We presented a novel method to cluster and order genetic markers from genotyping data obtained from several population types including doubled haploid, backcross, haploid and recombinant inbred line. The method is based on solid theoretical foundations and as a result is computationally very efficient. It also gracefully handles missing observations and is capable of tolerating some genotyping errors. The proposed method has been implemented into a software tool named MSTMap, which is freely available in the public domain at http://www.cs.ucr.edu/~yonghui/mstmap.html. According to our extensive studies using simulated data, as well as results obtained using a real data set from barley, MSTMap outperforms the best tools currently available, particularly when the input data are noisy or incomplete.

The next computational challenge ahead of us involves the problem of integrating multiple maps. Nowadays, it is increasingly common to have multiple genetic linkage maps for the same organism, usually from a different set of markers obtained with a variety of genotyping technologies. When multiple genetic linkage maps are available for the same organism it is often desirable to integrate them into one single *consensus map*, which incorporates all the markers and ideally is consistent with each individual map. The problem of constructing a consensus map from multiple individual maps remains a computationally challenging and interesting research topic.

## Supporting Information

Text S1 Supplementary Text: Efficient and Accurate Construction of Genetic Linkage Maps.
(0.08 MB PDF)
