# Supplementary material for: Efficient and Accurate Construction of Genetic Linkage Maps from the Minimum Spanning Tree of a Graph
Source: PLoS Genet. 2008 Oct 10;4(10):e1000212. doi: 10.1371/journal.pgen.1000212 (PMC2556103; doi:10.1371/journal.pgen.1000212)
Supplement: Text S1 — Supplementary Text: Efficient and Accurate Construction of Genetic Linkage Maps. (0.08 MB PDF) [file pgen.1000212.s001.pdf]

Efficient and accurate construction of genetic linkage maps: Supplementary Text S1

Yonghui Wu[1], Prasanna R. Bhat[2], Timothy J. Close[2] and Stefano Lonardi[1]

[1] Department of Computer Science and Engineering, University of California, Riverside, CA, 92521, USA

[2] Department of Botany and Plant Sciences, University of California, Riverside, CA, 92521, USA

1 Theorems and Proofs

Theorem 1. *Let l_i and l_j be two markers that belong to two different LGs, and let $d_{i,j}$ be the Hamming distance between $\mathbb{A}[i,]$ and $\mathbb{A}[j,]$. Then,*

$$E(d_{i,j}) = n/2 \quad \textit{and} \quad \mathbf{P}(d_{i,j} < \delta) \leq e^{-\frac{2(n/2-\delta)^2}{n}}$$

where $\delta < n/2$.

Proof. Let $c_k \in N$ and let $X_{i,j}^k$ be a random indicator variable which is equal to 1 if c_k is a recombinant with respect to l_i and l_j and to 0 otherwise. Clearly $E(X_{i,j}^k) = \frac{1}{2}$, and $d_{i,j} = \sum_k X_{i,j}^k$. The family of random variables $\{X_{i,j}^k : 1 \leq k \leq n\}$ are i.i.d. According to linearity of expectation, $E(d_{i,j}) = n/2$. The bound $\mathbf{P}(d_{i,j} < \delta) \leq e^{-\frac{2(n/2-\delta)^2}{n}}$ derives directly from Hoeffding's inequality [1]. □

In the rest of this Section, let us assume that all the markers in M belong to the same linkage group. Let $G(M, E)$ be an edge-weighted complete undirected graph on the set of vertices M, and let w be the associated semi-linear weight function. Let Π be an order of the markers in M. The weight of Π, which is denoted as $w(\Pi)$, is defined as the weight of the TSP of G corresponding to Π. We have the following Lemma.

Lemma 1. *Let Π_0 be the true order of the markers (according to their positions on the chromosome). If w is semi-linear, then $w(\Pi_0)$ is minimal among all the possible orders of the markers.*

Proof. Let $\Pi_0 = l_1, l_2, \ldots, l_m$ be the true order of the markers in M, and let $\Pi_1 = l_{t_1}, l_{t_2}, \ldots, l_{t_m}$ be any order. Let $P_0 = \{(l_{i-1}, l_i) | 2 \leq i \leq m\}$ be the set of consecutive marker pairs in Π_0 and $P_1 = \{(l_{t_{j-1}}, l_{t_j}) | 2 \leq j \leq m\}$ be the set of consecutive marker pairs in Π_1. P_0 and P_1 each contains $m-1$ marker pairs. Let $G(P_0, P_1, E)$ be a bipartite graph where there is an edge between

vertex $(l_{i-1}, l_i) \in P_0$ and vertex $(l_{t_{j-1}}, l_{t_j}) \in P_1$ if (l_{i-1}, l_i) is enclosed in $(l_{t_{j-1}}, l_{t_j})$. According to the construction of $G(P_0, P_1, E)$, any vertex $v \in P_1$ is mapped to one or more vertices in P_0, because the pair $(l_{t_{j-1}}, l_{t_j})$ corresponding to v must enclose one or more marker pairs in P_0 and Π_0 is the true order of the markers. Furthermore, any subset $T \subseteq P_1$ is mapped to a subset $S \subseteq P_0$ with $|T| \leq |S|$. Therefore, according to the *Hall's Marriage Theorem* [2] there exists a perfect matching between P_0 and P_1. This means that there is an one to one correspondence between the elements in P_0 and the elements in P_1, such that if $(l_{i-1}, l_i) \in P_0$ is mapped to $(l_{t_{j-1}}, l_{t_j}) \in P_1$ then (l_{i-1}, l_i) is enclosed in $(l_{t_{j-1}}, l_{t_j})$. Accordingly $w(i-1, i) \leq w(t_{j-1}, t_j)$ since w is semi-linear. Therefore, we conclude that $w(\Pi_0) = \sum_{2 \leq i \leq m} w(i-1, i)$ is less than or equal to $w(\Pi_1) = \sum_{2 \leq j \leq m} w(t_{j-1}, t_j)$. □

According to Lemma 1, in order to determine the correct order of the markers, one has to find the minimum weight TSP in G under a proper weight function w. Although the problem of finding the minimum weight TSP in a general graph is NP-complete [3], in our case the problem is much easier as shown next. A *minimum (weight) spanning tree* (MST) of G is a subgraph of G which is a tree that spans all the vertices of G and has minimum total weight. To be technically accurate, we assume that the graph G has exactly one minimum weight spanning tree.

Lemma 2. *Let $G(M, E)$ be the weighted complete graph on the markers M. Suppose that the weight function w on the edges of G is semi-linear, and that the MST Γ_0 for G is unique. Let Γ_0 be such MST, then Γ_0 is the minimum weight TSP of G.*

Proof. Let $l_1, l_2, \ldots, l_m$ be the markers in their correct order. Let us run Prim's minimum spanning tree algorithm [4] on G starting from the first marker in the linkage group, i.e., l_1. Prim's algorithm iteratively adds node (and edges) to a partially discovered tree until all the nodes are included. The next node to be added is the closest one to the partially discovered tree. Let l_{i-1} be the node added in the previous step of Prim. Given that w is semi-linear, the next marker to be added will be l_i. Therefore, the MST is also a TSP in G.

Due to the fact that the weight of the MST is the lower-bound on the weight of the optimal TSP, the TSP identified via Prim's algorithm is indeed the minimum weight TSP. □

In order for Lemma 2 to hold, the MST must be unique. A sufficient (but not necessary) condition for the MST to be unique is that all weights are distinct. A common situation in practice is to have several edges with zero weight indicating co-segregating markers, which could lead to non-unique MSTs. In practice, due to limited sample size and low recombination rate, co-segregating markers are common. This problem can be easily dealt with by first collecting the co-segregating markers together and then arbitrarily choosing one marker as the representative. The mapping procedure is carried out only on the representative markers. By construction, pairwise weights between representative markers are strictly positive. Since there is no information to infer the relative orders between co-segregating markers, non-representative markers will be mapped to the same position as the corresponding representatives.

Theorem 2. *Let M be a set of representative markers, and $G(M, E)$ be the corresponding complete weighted graph, where edges are weighted according to a semi-linear function w. If G has a unique MST T, then T indicates the correct order of the marker bins.*

Proof. Follows directly from Lemma 1 and Lemma 2. □

2 Pseudocode of the algorithms

Algorithm 1 ORDER(G)

$G = (B, E)$ is an edge-weighted complete graph corresponding to some linkage group. B is the set of bins/markers in the linkage group.

$T_0 \leftarrow$ Run Prim's MST algorithm on G
$\Pi_0 \leftarrow$ Compute the backbone of T_0
if $(|\Pi_0| = |B|)$ **then** {*the MST is a path*}
  return Π_0
else
  $S \leftarrow B - \Pi_0$ {*get the off-the-backbone bins*}
  for $l_i \in S$ **do** {*construct the initial map*}
    $\Pi_0 \leftarrow$ insert l_i to its optimal position in Π_0
  while improvement **do** {*improve* Π_0 *to a local optima*}
    K-opt(Π_0)
    node-relocation(Π_0)
    block-optimize(Π_0)
  return Π_0

Algorithm 2 EM($\mathbb{A}$, S)

$\mathbb{A}$: the probability matrix corresponding to some linkage group. $\mathbb{A}[i,j] = 1$ if the corresponding genotype is `A` and $\mathbb{A}[i,j] = 0$ if the corresponding genotype is `B`. $\mathbb{A}[i,j] = 0.5$ if $(i,j) \in S$, that is $\mathbb{A}[i,j]$ is a missing observation.
S: the list of missing observations on the linkage group

Construct $G(B, E)$. Assign the initial weights for edges in E by ignoring the missing observations.
$\Pi_0 \leftarrow$ call ORDER(G) to get the initial order of the markers {*the initialization step*}
repeat {*EM algorithm*}
  adjust $\mathbb{A}[i,j]$ for those missing observations in S according to Equation (1) in Main Text {*E-step*}
  $\Pi_0 \leftarrow$ recompute $d_{i,j}$ according to Equation (2) in Main Text and call ORDER(G) to get a refined map {*M-step*}
until Π_0 converges
return $\Pi_0, d_{i,j}$ {*return the final map as well as the final pairwise distances*}

Algorithm 3 MSTMAP($\mathbb{A}$, S, ϵ)

$\mathbb{A}$: the probability matrix containing raw data for all the markers
S: the list of missing observations
ϵ: the parameter for clustering (default is 0.00001)

Compute the pairwise distances $d_{i,j}$ for all i, j according to Equation (2)
Construct the weighted complete graph $G(M, E)$, where the weight edge (l_i, l_j) is $d_{i,j}$
Solve for δ from the equation $-2(n/2 - \delta)^2/n = \log_e \epsilon$
$E \leftarrow E - \{(l_i, l_j) \in E | d_{i,j} \geq \delta\}$ {*remove inter-LG edges from* E}
$CC \leftarrow BFS(G)$ {*get the connected components in G*}
for all linkage group $g \in CC$ **do** {*iterate through each linkage group*}
    Let $\mathbb{A}_g$ be a subset of $\mathbb{A}$ corresponding markers in g
    Let S_g be a subset of S corresponding to the missing observations in linkage group g
    $T_g \leftarrow \emptyset$ {T_g *is the set of suspicious observations have been identified so far*}
    $X \leftarrow \infty$ {X *is the sum of the total number of suspicious observations and the total number of cross-overs. It is initialized as* ∞.}
    while $(true)$ **do** {*repeatedly detect errors and refine the map*}
        $\Pi_g, d_{i,j} \leftarrow$ EM(A_g, $S_g \cup T_g$) {*call* EM *to get a refined map as well as the pairwise distance based on the latest estimation of missing and suspicious data*}
        $X' \leftarrow$ (the number cross-overs in $\Pi_g) + |T_g|$
        if $X' < X$ **then**
            $X \leftarrow X'$
            $T' \leftarrow \emptyset$
            for all (i, j) not in $S_g \cup T_g$ **do**
                $\mathbb{E}\mathbb{A}_g[i, j] \leftarrow$ compute $\mathbb{E}\mathbb{A}_g[i, j]$ according to Equation (3) in Main Text
                if $|\mathbb{E}\mathbb{A}_g[i, j] - \mathbb{A}_g[i, j]| > 0.75$ **then**
                    $T' \leftarrow T' \cup \{(i, j)\}$
            $T_g \leftarrow T_g \cup T'$
        else
            break
return $\{\Pi_g | g \in CC\}$ {*return the collection of final maps for all the linkage groups*}

3 Extension to RIL population

The various types of genetic mapping populations can be divided into two general classes according to the number of possible distinct genotype states that may arise. For the first class, at any locus of interest there can be only two distinct genotype states (donated as `A` and `B` respectively, missing is not counted as a distinct genotype state). Mapping populations that fall into this class include DH, Hap, BC1 and advanced RIL, among others. For the second class, at any locus of interest there can be three distinct genotype states (denoted as `A`, `B` and `AB` respectively. Again missing is not counted as a distinct genotype state). This class includes population types such as F2 and less advanced RIL. In general, the first class is preferred in genetic mapping due to its simplicity.

In our discussion so far, we have been focusing on the first class of populations, but our algorithm can be extended to the second class as well. As shown in Section 1, in order to find the true order of the markers we need a weight function that is semi-linear. Our weight function is the pairwise recombination probability $\mathbf{P}_{i,j}$. The maximum likelihood estimates for $\mathbf{P}_{i,j}$ for the first class of population are $\hat{\mathbf{P}}_{i,j} = d_{i,j}/n$ where $d_{i,j}$ is the pairwise distance between marker l_i and l_j. In the following, we show how $\mathbf{P}_{i,j}$ can be estimated for a RIL population at generation r.

A RIL population at generation r is obtained by first crossing two fully homozygous parents to obtain an F1 population, followed by repeatedly selfing with single seed descendant for $r-1$ generations [5]. The three possible genotype states in an RIL population are `A`, `B` and `AB`. It can be shown that the expected fraction of heterozygous `AB` in an RIL population at generation r is $2^{-(r-1)}$. When r is large, the proportion of `AB` states are negligible, and hence we can treat advanced RIL as a DH population. If r is small, we have to deal with `AB` explicitly, as follows.

For two loci l_1 and l_2 of interest, we denote their status using the following notation $\frac{ab}{cd}$, where a represents the state of locus l_1 on the paternal chromosome, b represents the state of locus l_2 on the paternal chromosome, c represents the state of locus l_1 on the maternal chromosome, and d represents the state of locus l_2 on the maternal chromosome. The ten possible zygotic types are divided into five categories. We have $C = \{\frac{\mathtt{AA}}{\mathtt{AA}}, \frac{\mathtt{BB}}{\mathtt{BB}}\}$, $D = \{\frac{\mathtt{AB}}{\mathtt{AB}}, \frac{\mathtt{BA}}{\mathtt{BA}}\}$, $E = \{\frac{\mathtt{AB}}{\mathtt{AA}}, \frac{\mathtt{BB}}{\mathtt{BA}}, \frac{\mathtt{BA}}{\mathtt{AA}}, \frac{\mathtt{BB}}{\mathtt{AB}}\}$, $F = \{\frac{\mathtt{AA}}{\mathtt{BB}}\}$ and $G = \{\frac{\mathtt{AB}}{\mathtt{BA}}\}$.

Let C_i, D_i, E_i F_i and G_i be the proportion of individuals in an RIL population at the ith generation having zygotic types C, D, E, F and G respectively. According to Haldane and Waddington [6] C_{i+1}, D_{i+1}, E_{i+1} F_{i+1} and G_{i+1} can be computed from C_i, D_i, E_i F_i, G_i and the per meiosis

recombination probability p between l_1 and l_2 as follows

$$\begin{aligned}
C_{n+1} &= C_n + \frac{1}{4}E_n + \frac{1}{2}(1-p)^2 F_n + \frac{1}{2}p^2 G_n \\
D_{n+1} &= D_n + \frac{1}{4}E_n + \frac{1}{2}p^2 F_n + \frac{1}{2}(1-p)^2 G_n \\
E_{n+1} &= \frac{1}{2}E_n + 2p(1-p)(F_n + G_n) \\
F_{n+1} &= \frac{1}{2}(1-p)^2 F_n + \frac{1}{2}p^2 G_n \\
G_{n+1} &= \frac{1}{2}p^2 F_n + \frac{1}{2}(1-p)^2 G_n
\end{aligned} \tag{1}$$

It can be easily verified that $C_{i+1} + D_{i+1} + E_{i+1} + F_{i+1} + G_{i+1} = C_i + D_i + E_i + F_i + G_i$. At generation 1, i.e., in the F1 generation, we have $C_1 = 0.0$, $D_1 = 0.0$, $E_1 = 0.0$, $F_1 = 1.0$ and $G_1 = 0.0$. Given p and r, we compute the expected values for C_r, D_r, E_r, F_r and G_r using Equations (1). However, our problem is the inverse. The experimental data gives C_r, D_r, E_r and $F_r + G_r$, and the problem is to find a p such that the expected fractions for each category is close to the observed fractions for each category. Our approach is to break the interval $(0, 0.5)$ into small enough subintervals, and find the value of p such that the sum of square errors between the observed fractions and the expected fractions is minimized. Once all the pairwise recombination probabilities have been estimated, we use Algorithm 1 to find the order of the markers.

References

[1] Hoeffding W (1963) Probability inequalities for sums of bounded random variables. Journ Am Stat Ass 58:13–30.

[2] Hall P (1935) On representatives of subsets. J London Math Soc s1-10:26–30.

[3] Garey M, Johnson D (1979) Computers and Intractability: A Guide to the Theory of NP-Completeness. New York: WH Freeman and Company.

[4] Cormen TH, Leiserson CE, Rivest RL, Stein C (2001) Introduction to Algorithms, Second Edition. The MIT Press and McGraw-Hill Book Company.

[5] Broman KW (2005) The genomes of recombinant inbred lines. Genetics 169:1133–1146.

[6] Haldane JBS, Waddington CH (1931) Inbreeding and linkage. Genetics 16:357–374.
